# Nonannual tree rings in a climate‐sensitive *Prioria copaifera* chronology in the Atrato River, Colombia

**DOI:** 10.1002/ece3.2905

**Published:** 2017-07-06

**Authors:** David Herrera‐Ramirez, Laia Andreu‐Hayles, Jorge I. del Valle, Guaciara M. Santos, Paula L. M. Gonzalez

**Affiliations:** ^1^ Maestría en Bosques y Conservación Ambiental Universidad Nacional de Colombia Sede Medellín Medellin Colombia; ^2^ Tree‐Ring Laboratory Lamont‐Doherty Earth Observatory of Columbia University Palisades NY USA; ^3^ Institut Català de Ciències del Clima (IC3) Barcelona Catalonia Spain; ^4^ Department of Earth System Science University of California Irvine CA USA; ^5^ International Research Institute for Climate and Society Columbia University Palisades NY USA

**Keywords:** ^14^C, annual and subannual tree rings, climate signals, El Niño South Oscillation, sea surface temperature, streamflow, tropical dendrochronology

## Abstract

In temperate climates, tree growth dormancy usually ensures the annual nature of tree rings, but in tropical environments, determination of annual periodicity can be more complex. The purposes of the work are as follows: (1) to generate a reliable tree‐ring width chronology for *Prioria copaifera* Griseb. (Leguminoceae), a tropical tree species dwelling in the Atrato River floodplains, Colombia; (2) to assess the climate signal recorded by the tree‐ring records; and (3) to validate the annual periodicity of the tree rings using independent methods. We used standard dendrochronological procedures to generate the *P. copaifera* tree‐ring chronology. We used Pearson correlations to evaluate the relationship of the chronology with the meteorological records, climate regional indices, and gridded precipitation/sea surface temperature products. We also evaluated 24 high‐precision ^14^C measurements spread over a range of preselected tree rings, with assigned calendar years by dendrochronological techniques, before and after the bomb spike in order to validate the annual nature of the tree rings. The tree‐ring width chronology was statistically reliable, and it correlated significantly with local records of annual and October–December (OND) streamflow and precipitation across the upper river watershed (positive), and OND temperature (negative). It was also significantly related to the Oceanic Niño Index, Pacific Decadal Oscillation, and the Southern Oscillation Index, as well as sea surface temperatures over the Caribbean and the Pacific region. However, ^14^C high‐precision measurements over the tree rings demonstrated offsets of up to 40 years that indicate that *P. copaifera* can produce more than one ring in certain years. Results derived from the strongest climate–growth relationship during the most recent years of the record suggest that the climatic signal reported may be due to the presence of annual rings in some of those trees in recent years. Our study alerts about the risk of applying dendrochronology in species with challenging anatomical features defining tree rings, commonly found in the tropics, without an independent validation of annual periodicity of tree rings. High‐precision ^14^C measurements in multiple trees are a useful method to validate the identification of annual tree rings.

## 1 INTRODUCTION

Dendrochronology is a powerful, inexpensive, and relatively fast methodology used to study ecology and growth of tree species that form annual rings, as well as to extend climate records where instrumental data are scarce (Fritts, 1976). In temperate regions, due to a marked seasonality, most of the trees have well‐defined annual rings that allow for an extensive application of dendrochronology (Stokes & Smiley, 1996). Despite an increase in dendrochronological studies in the tropics (Rozendaal & Zuidema, 2011; Villalba et al., 2011), major efforts are still needed for its widespread application, due to the challenges of identifying ring boundaries defining the annual nature of tree rings that is essential for developing reliable chronologies, among other factors. The crossdating technique, a fundamental dendrochronological principle, is the matching of growth patterns between tree‐ring samples from the same tree and between trees in a given area, which guarantees that the absolute calendar year is assigned to each individual tree ring. While in temperate regions statistically significant crossdating has been accepted as sufficient proof for the identification of annual rings (Stahle, Mushove, Cleaveland, Roig, & Haynes, 1999), in the tropics the annual periodicity of tree layers cannot be taken for granted.

The development of long and reliable tropical chronologies has been hampered by several issues such as indistinct growth rings (Groenendijk, Sass‐Klaassen, Bongers, & Zuidema, 2014; Wils, Robertson, Eshetu, Sass‐Klaassen, & Koprowski, 2009); partially absent (i.e., “missing”), false, and/or wedging rings (Rozendaal & Zuidema, 2011; Worbes, 2002); crossdating problems (Wils et al., 2009); lack of significant relationships with climate (Trouet, Esper, & Beeckman, 2010); lack of information regarding tropical tree ages (López & Villalba, 2011; Worbes, 1999); and high decay rate of dead wood (Trouet et al., 2010). These kind of issues have been associated with drought stress, growth‐suppressing conditions, intense competition (Schweingruber, 1996), flooding (Junk, Piedade, Wittmann, Schöngart, & Parolin, 2010), and/or changes in topography (López & Villalba, 2011). In these cases, ring counts at the stem base or the apparent root collar often did not reveal the true age of individuals (López & Villalba, 2011; Wils, Sass‐Klaassen, et al., 2011). These challenges obstruct the identification of annual growth layers in the tropics, which is fundamental to developing reliable tree‐ring chronologies and climate reconstructions for advances in tree‐ring research globally. Nonetheless, even in wet tropical forests, evidences of annual tree‐ring formation in several tropical species have been reported by successful crossdating and correlation with climate data (Soliz‐Gamboa et al., 2011; Worbes, 1999) and ^14^C bomb peak dating (Groenendijk et al., 2014; Soliz‐Gamboa et al., 2011; Wils, Robertson, et al., 2011; Worbes & Junk, 1989).

Bomb peak dating using ^14^C has been successfully conducted not only in temperate (Biondi, Strachan, Mensing, & Piovesan, 2007; Pearson, Hua, Allen, & Bowman, 2011) and subtropical regions (Biondi & Fessenden, 1999), but also in trees and shrub species from the tropics (Fichtler, Clark, & Worbes, 2003; Groenendijk et al., 2014; Worbes & Junk, 1989). Nevertheless, calendar year misidentifications using ^14^C bomb peak dating have been also reported in tree species such as *Brachystegia cynometroides*,* Brachystegia eurycoma*, and *Daniellia oge* in Africa (Groenendijk et al., 2014); *Clarisia racemosa Peltogyne* cf.*heterophylla* and *Cedrelinga catenaeformis* in Bolivia (Soliz‐Gamboa et al., 2011); subtropical *Callitris endlicheri* and the temperate species *Callitris glaucophylla* in Australia (Pearson et al., 2011). Overall, radiocarbon dating provides an independent validation of the frequency of tree‐ring formation for the recent past (e.g., for the last 60 years) and allows testing the synchrony of ring patterns when rings from several years are analyzed. The dendrochronological method allows assigning calendar dates to the tree rings by crossdating tree‐ring series from a particular region. The radiocarbon method synchronizes ^14^C signatures embedded in individual tree rings to those of the atmosphere obtained from samples of ambient air and/or tree rings around the globe during the bomb peak period (e.g., from 1955 to today). Once crossdated growth rings of a sample and its ^14^C signature yield the same calendar age, one can be sure that the dendrochronological ages obtained are correct.

**Figure 1:**
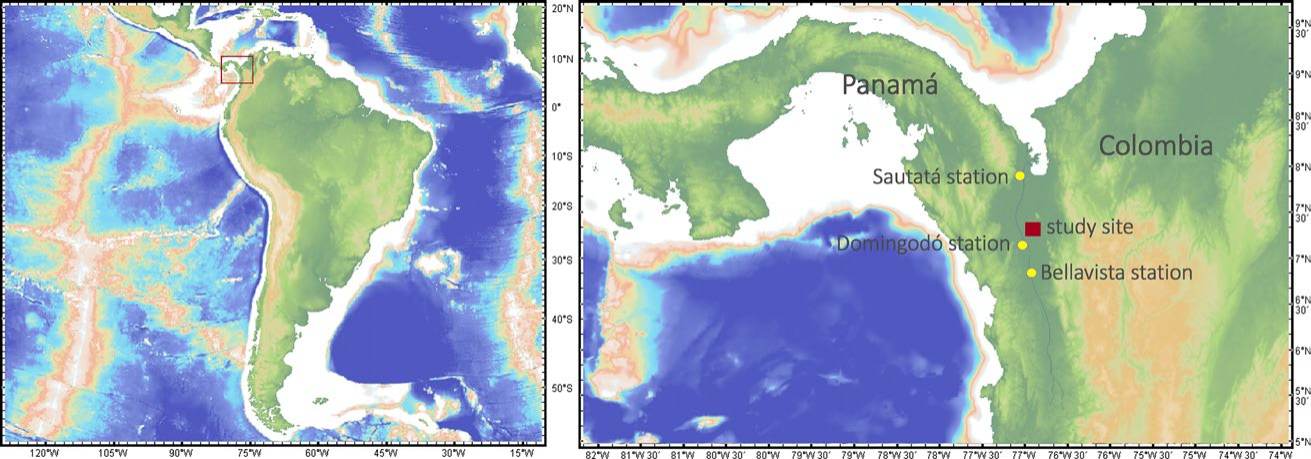
Study site in the low Atrato River (7°20′N, 76°57′W). Yellow points show hydroclimatic stations: Domingodó Station (77°02′N, 7°10′W; precipitation and water levels; 1967–2006), Sautatá Station (77°07′N, 7°51′W; temperature; 1972–2005), and Bellavista Station (76°57′N, 5°57′W; streamflow; 1965–2002). This map was generated using GeoMapApp (URL: http://www.geomapapp.org) and the Global Multi‐Resolution Topography (Ryan et al. 2009)

*Prioria copaifera* Griseb. (Leguminoceae) is an evergreen species that dwells in the Atrato River floodplains (Figure 1). In these environments *P. copaifera* trees are exposed to high flooding levels over more than 8 months per year, with a shorter period of low streamflow. These interannual streamflow fluctuations can be an environmental pace‐marker leading to interannual tree growth variations (Grauel, 2004; Herrera & del Valle, 2011). *Prioria copaifera* growth and ecology has been relatively well studied due to its commercial value, but its phenology and climatic relationships are still unknown (del Valle, 1986; Giraldo & del Valle, 2011; Grauel, 2004). This work will evaluate the annual nature and the climate signal of tree rings in *P. copaifera* by: (1) generating a reliable tree‐ring width chronology from trees located at the low Atrato River in northwestern Colombia using standard dendrochronological techniques; (2) assessing the climate signal recorded by the tree‐ring records; (3) validating the annual periodicity of the tree rings using ^14^C analyses.

## 2 MATERIALS AND METHODS

### 2.1 Site description and climate variability

**Figure 2:**
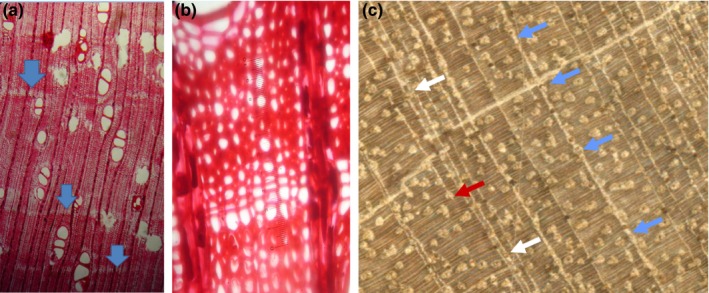
Anatomical detail of *P. copaifera* tree rings: A) image at 75x B) image at 100x, and C) image at 40x. Blue arrows show the tree ring identified as real, white arrows show wedging rings and red arrow shows false rings

We collected 15 cross sections from *P. copaifera* trees in the low Atrato River in Colombia (7°20′N, 76°57′W; Figure 1). *Prioria copaifera* does not show a clear transition from sapwood to heartwood and exhibits a diffuse‐porous wood anatomy. The boundaries between growth layers are defined by marginal parenchyma, without a distinct earlywood–latewood transition (Figure 2). The vessels are diffuse, solitary or in radial groups of 2–3 vessels, medium size (132 ± 25 μm), and just visible to the naked eye with less than five vessels per mm^2^ (López, del Valle, & Giraldo, 2014).

**Figure 3:**
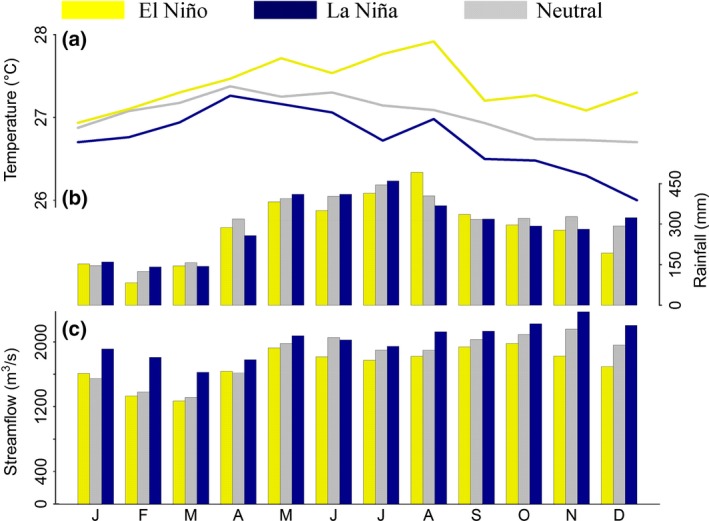
Seasonal cycle of temperature (a), precipitation (b), and streamflow (c) for distinct El Niño South Oscillation (ENSO) phases: positive (El Niño), negative (La Niña), and neutral

The hydroclimatic records are from the Colombian Institute of Hydrology, Meteorology, and Environmental Studies (IDEAM, Table 1). The seasonal cycle of precipitation shows higher precipitation from May to November and lower precipitation from December to April, whereas high temperatures last from March to August, and lower temperatures persist from September to February (Figure 3a). The amplitude of the seasonal cycle for temperatures is 1°C and ranges from 26.5 to 27.5°C. Precipitation can reach 3574 mm on average per year and ranges from 2268 to 4659 mm (Figure 3b). The streamflow variability drives flooding patterns in the low Atrato River keeping the floodplains under water from May to December reaching up to 3000 m^3^/s in November–December, whereas from February to April decreases in the river water levels are experienced (Figure 3c). The climate variability over this watershed is also strongly affected by El Niño‐Southern Oscillation (ENSO), showing lower precipitation and streamflow amounts during El Niño years and higher during La Niña years (Figure 3).

**Table 1:** Mean Pearson correlation products (*r*) of the *Prioria copaifera* tree‐ring width chronology with the annual instrumental records (total precipitation, mean temperature, mean water levels, and mean streamflow) and annual climate indices such as the Oceanic Niño (ONI), the Pacific Decadal Oscillation (PDO), the Southern Oscillation Index (SOI), and the North Atlantic Ocean (NAO)

Source	Coordinates	Climatic variable/indices	*r*_1_	Time span_1_	*r*_2_	Time span_2_
Domingodó Station	77°02′N, 7°10′W	Annual precipitation	.06	1967–2006	−.06	1980–2006
Sautatá Station	77°07′N, 7°51′W	Annual mean temperature	−.22	1972–2005	−.23	1980–2005
Domingodó Station	77°02′N, 7°10′W	Annual mean water levels	.31	1979–2003	.3	1980–2003
Bellavista Station	76°57′N, 5°57′W	Annual mean streamflow	.40*	1965–2002	.51*	1980–2002
NOAA		ONI index	−.25*	1950–2006	−.39*	1980–2006
NOAA		SOI index	.14	1951–2006	.26*	1980–2006
NOAA		PDO index	−.12	1948–2006	−.26*	1980–2006
NOAA		NAO index	.02	1950–2006	.09	1980–2006

Correlations were conducted for the maximum time span available for each record (*r*_1_) and for a shorter period from 1980 to 2006 (*r*_2_). Significant correlations are indicated by * (*p* < .05).

### 2.2 Sample description and dendrochronological procedure

The sampled cross sections were analyzed at the Forest and Climate Change Laboratory at the *Universidad Nacional de Colombia* at Medellín, and a preliminary tree‐ring width chronology using 10 of the 15 trees was developed (Herrera & del Valle, 2011). In three trees, ^14^C measurements were taken on wood from the ring of the 1994 calendar year for assessing the annual periodicity of the growth bands. Here, we re‐processed, re‐crossdated, and re‐measured the 10 samples used by Herrera and del Valle (2011) and added five samples in order to attain a robust chronology. The samples were progressively polished with 80 to 2,000 grain sandpaper. To unambiguously mark the tree rings, we evaluated the continuity of the growth layers around the entire circumference and the wedging rings were marked as true rings, identifying when the tree rings were locally mismatched or absent. Discontinuous parenchyma bands identified false rings. A total of 31 ring‐width series resulted from measuring 2 or 3 radii from each of the 15 cross sections collected. The visual crossdating was performed by matching tree growth patterns within individual (from the same tree) and among individuals (from different trees) under a Meiji stereo zoom at 40× magnification using the Yamaguchi method (Yamaguchi, 1991). The ring widths were measured over a straight pathway from pith to bark on scanned images of each cross section using the software WinDendro (Version 2009b, Regent Instruments, Canada). For scanning, we used a resolution of 2400 dpi on a flatbed scanner calibrated by Regent Instruments (Epson Expression 10000XL). This guaranteed a precision of 0.01 mm in the measurements. The crossdating was validated using the statistical software COFECHA (Grissino‐Mayer, 2001; Holmes, 1983) and the dplR package in R software (Bunn, 2008) that identifies possible errors using correlations by running segments between individual tree‐ring series and the master chronology. After this validation, the ring‐width raw individual series were standardized using a cubic spline with a 50% frequency response at 60 years and an autoregressive model was applied to remove the autocorrelation. The resulting individual residual series were used to build a mean chronology calculated with a biweight robust mean (Cook & Kairiukstis, 1990) using dplR (Bunn, 2008, 2010; Bunn et al., 2017; R Core Team 2013).

### 2.3 Relationships with climate

We used correlation analysis, conducted with the bootres package in R (Zang & Biondi, 2013), to evaluate the monthly and seasonal relationship between the developed residual chronology without autocorrelation and local climate and regional climate indices. The significance of correlations was evaluated with a 95% confidence interval from nonparametric test based on bootstrapping. Only time series that overcame normality, trend, and homogeneity tests were used. The climate variables were local precipitation, temperature, river water levels, and streamflow from the local meteorological stations (Table 1, Figure 1), as well as regional climate indices associated with ocean and atmospheric dynamics such as the Oceanic Niño Index (ONI), the Pacific Decadal Oscillation (PDO), and the Southern Oscillation Index (SOI). The North Atlantic Oscillation (NAO) was also included. However, the NAO index should not show any significant correlation with our chronology as its impacts are mainly centered on the Northern Hemisphere. We also used gridded precipitation data from GPCC V6 0.5° (Schneider et al., 2011) and HaddlSST1 1° sea surface temperature (SST; Kaplan et al., 1998) to evaluate the spatial relationship of gridded precipitation and SST with streamflow records and the residual tree‐ring width chronology. The spatial correlations were calculated monthly, annually, and seasonally. The significance of the correlations was evaluated using a nonparametric 90% confidence interval, and only coefficients with *p *<* *.10 level were plotted in the maps.

### 2.4 Radiocarbon dating

The application of ^14^C bomb peak dating is based on the excess of ^14^C in the atmosphere caused by aboveground atomic weapons tests mostly concentrated between 1954 and 1963 (Groenendijk et al., 2014; Hua, Barbetti, & Rakowski, 2013). Here, we selected three different trees to verify the annual pattern of tree‐ring formation when applying the ^14^C bomb peak dating method coupled with high‐precision accelerator mass spectrometry (AMS; Beverly et al., 2010). The tree termed Pg25 was randomly chosen, while the trees Pg20 and Pg11 were preselected due to their high correlation with the master chronology. In each tree, we tested eight tree rings spaced about 10 rings apart to reproduce the ^14^C bomb spike in the tropics around the calendar year of 1965. By analyzing samples from single preselected dendrochronological calendar years roughly spaced before and shortly thereafter of the ^14^C bomb spike, it is possible to identify potential errors in crossdating and ring identification as well as any other ambiguities in the ^14^C sample processing and measurements (Andreu‐Hayles et al., 2015). While discrepancies in the dendrochronological calendar dating can be attributed to the presence of partial absent rings or false rings as a result of opportunistic growth not necessarily annual, problems in ^14^C sample processing and measurement errors are normally associated with insufficient sample cleaning or contamination, or poor spectrometer performance. The ^14^C problems (if any) can be addressed by processing and measuring reference materials alongside with the samples of interest (Santos, Linares, Lisi, & Tomazello Filho, 2015). If those reference material samples are in concordance with the expected consensus values, age offsets are likely to be due to other factors undetected by the dendrochronological approach.

Nevertheless, if all dates coincide between dendrochronological dated rings and ^14^C measurements (margin error ±1 year), then the rings were formed annually and no dating or measurement errors occurred. From the 24 rings selected, we collected 35–50 mg of wood to determine ^14^C concentrations. A holocellulose fraction was then isolated from the original wood samples by temperature‐controlled aqueous baths of acid and alkaline solutions followed by sodium chlorite treatment, as detailed in Andreu‐Hayles et al. (2015). Holocellulose extracts were converted to graphite targets, and then measured by ^14^C‐AMS technology. High‐precision ^14^C‐AMS measurements were performed at the Keck Carbon Cycle accelerator mass spectrometry (KCCAMS) facility (Beverly et al., 2010). The ^14^C data were mass‐dependent isotopic fractionation corrected using the online δ^13^C values measured by the spectrometer. Wood blank and reference samples subjected to the same holocellulose extraction procedure were also analyzed alongside with tropical tree‐ring samples to aid on accuracy and precision, following established protocols (Santos et al., 2010). Measured ^14^C values are given using the fraction modern carbon (Fm^14^C) notation (Stuiver & Polach, 1977). Uncertainties smaller than 0.3% were calculated based upon counting statistics, spectrometer isotopic fractionation and background corrections, and the scatter of results from primary and secondary standards, following the data analysis described in Santos et al. (2007). Finally, we compare the Fm^14^C (±*SD*) value of the samples with the ^14^C calibration curve NH‐zone 2 (Hua et al., 2013), which better represents the atmospheric ^14^C levels of the Northern Hemisphere in the area where the Atrato River site is located.

### 2.5 Assessment of the stability between climate and the tree‐ring records

We implemented a stochastic response function model based on the use of a submodel from structural time series models (STMs; Visser, Büntgen, D'Arrigo, & Petersen, 2010). This analysis allows us to verify the stability of the relationship between the ring‐width chronology and (1) the streamflow records (Bellavista station 1107701) from 1965 to 2002 and (2) the ONI index from 1950 to 2006. The methodology proposed by Visser et al. (2010) follows the model:

$$I_{t}\;=\;\mu_{t}\;+\;\alpha_{t}\;\times \;X_{t}\;+\;\varepsilon_{t},$$

where *I*_t_ stands for the ring‐width time series and *X*_t_ the climatic data. The “intercept” μ is traditionally a constant, but here it is replaced by a slowly bending trend model (μ_t_), the integrated random walk model. We replaced the constant response weight (α) by a stochastic counterpart α_t_ based on random walk models for individual climate variables. Both trend (μ_t_) and response weights (α) were estimated using the discrete Kalman filter (Harvey & Shephard, 1993; Visser & Molenaar, 1988); this filter is ideal in the sense that it yields the minimum mean square error estimates (normally distributed noise processes) for μ_t_ and α_t_ along with maximum‐likelihood estimates for unknown noise variances (Visser et al., 2010). The explained variance used here was computed as:

$${\text{Var}}_{\mathrm{explained}}\;=\;\left[1\;-\;\left(\frac{\text{var}(I_{t}\;-\;{\hat{\mu }}_{t}-{\hat{\alpha }}_{t}\;\times \;X_{t}}{\text{var}(I_{t}\;-\;{\hat{\mu }}_{t})}\right)\right]\;\times \;100,$$

where *I*_t_ is the ring‐width time series, *X*_t_ is the climatic variable, whereas ${\hat{\mu }}_{t}$ and ${\hat{\alpha }}_{t}$ are the estimated values for μ_t_ and α_t_, respectively. It can be understood as the measure of the explanatory power of adding *X*_t_ to the model, for example, how much of the phenomena is explained by adding an explanatory variable instead of only using the slowly bending trend *ȗ*_t_.

## 3 RESULTS

### 3.1 Tree‐ring chronology

**Figure 4:**
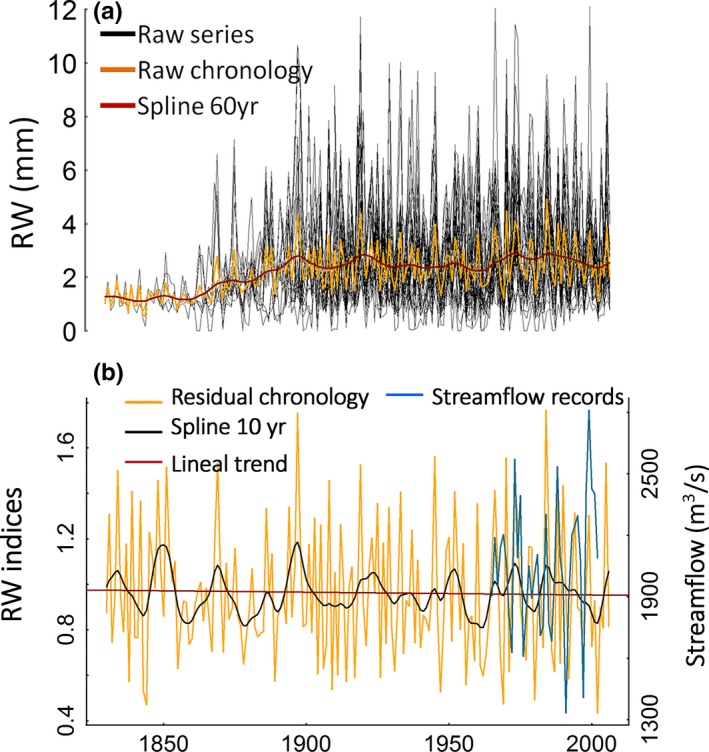
(a) 31 raw tree‐ring series from 15 trees of *Prioria copaifera*. (b) The *P. copaifera* residual tree‐ring chronology spanning from 1830 to 2006 overlapped with streamflow series from 1965 to 2002

The tree‐ring width chronology based on 31 series from 15 trees was successfully crossdated and spans from 1830 to 2006 (Figure 4). The mean correlation among the series was significant (*r *=* *.49, *p *<* *.01). The successful crossdating along the entire time span was supported by the finding of significant correlations between each series and the master series, performed over segments of 20 years lagged by intervals of 10 years. The resulting standard chronology exhibits a slightly significant first‐order negative autocorrelation (*r *=* *−.20, *p *<* *.05), which was removed in the residual chronology for the subsequent climate analysis. The chronology is statistically reliable from 1910 to 2006 based on the expressed population signal (EPS) that was above 0.85. This is an arbitrary threshold widely used by dendrochronological standard procedures to determine the reliability of tree‐ring chronologies (EPS > 0.85; Wigley, Briffa, & Jones, 1984).

### 3.2 Climate signals

**Figure 5:**
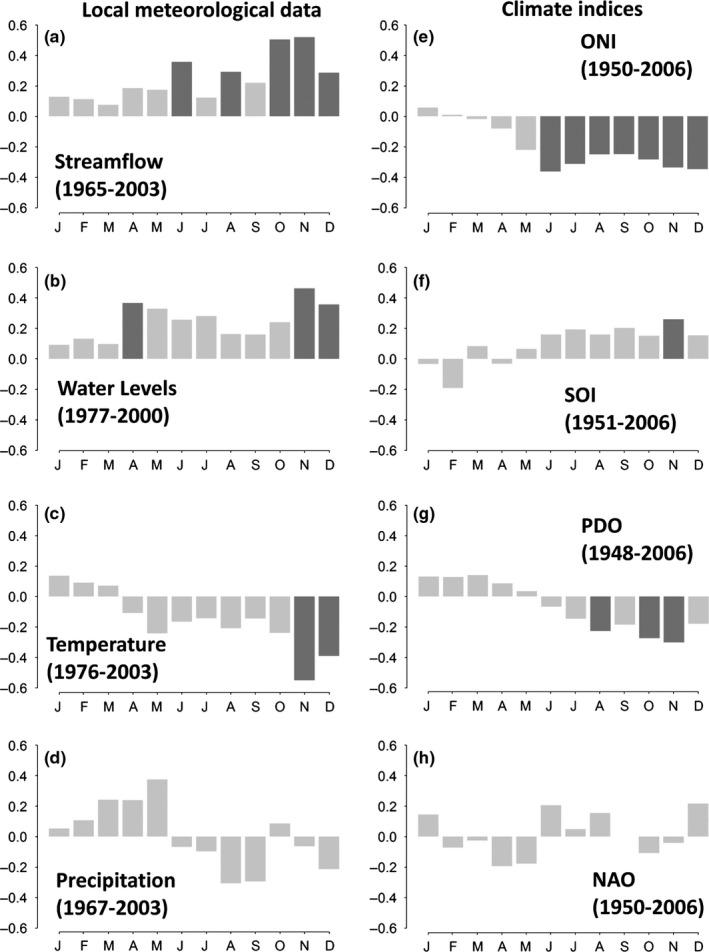
Boostrapped Pearson correlation analyses between the *Prioria copaifera* chronology and monthly climatic data: streamflow (a), water levels (b), temperature (c), and precipitation (d); as well as climatic indices: the Oceanic Niño Index, ONI (e), the Southern Oscillation Index, SOI (f), the Pacific Decadal Oscillation, PDO (g), and the North Atlantic Oscillation, NAO (h). Dark bars indicated significant correlations at *p* < .05 level

*Prioria copaifera* growth is significantly related to annual records of streamflow (positively) and ONI (negatively), while no significant relationships were found with annual precipitation and temperature series from the closest local meteorological station, nor SOI, PDO, and NAO (Table 1). Figure 5 illustrates that most of the significant monthly correlations occurred at the end of the rainy season. In agreement higher seasonal correlations were found during October–December (OND; Table 2) than annually (Table 1). Seasonally, *P. copaifera* growth is significant positively related with OND streamflow, November–December river water levels, and November SOI; negatively with November–December temperature, June to December ONI, October–November PDO, whereas no significant relationship was found with local precipitation nor NAO (Figure 5).

**Table 2:** Pearson correlation products (*r*) of the *Prioria copaifera* tree‐ring width chronology for the season October–November–December (OND) with instrumental records (total precipitation, mean temperature, mean water levels, and mean streamflow) and annual climate indices such as the Oceanic Niño (ONI), the Pacific Decadal Oscillation (PDO), the Southern Oscillation Index (SOI), and the North Atlantic Oscillation (NAO)

Source	Climatic variable/indices	*r*_1_	Time span_1_	*r*_2_	Time span_2_
Domingodó Station	OND precipitation	−.14	1967–2006	−.25	1980–2006
Sautatá Station	OND mean temperature	−.42*	1972–2005	−.47*	1980–2005
Domingodó Station	OND mean water levels	.36*	1979–2003	.35*	1980–2003
Bellavista Station	OND mean streamflow	.53*	1965–2002	.69*	1980–2002
NOAA	OND ONI index	−.30*	1950–2006	−.36*	1980–2006
NOAA	OND SOI index	.20	1951–2006	.23	1980–2006
NOAA	OND PDO index	−.28*	1948–2006	−.53*	1980–2006
NOAA	OND NAO index	.02	1950–2006	.20	1980–2006

Correlations were conducted for the maximum time span available for each record (*r*_1_) and for a shorter period from 1980 to 2006 (*r*_2_). Significant correlations are indicated by * (*p* < .05).

**Figure 6:**
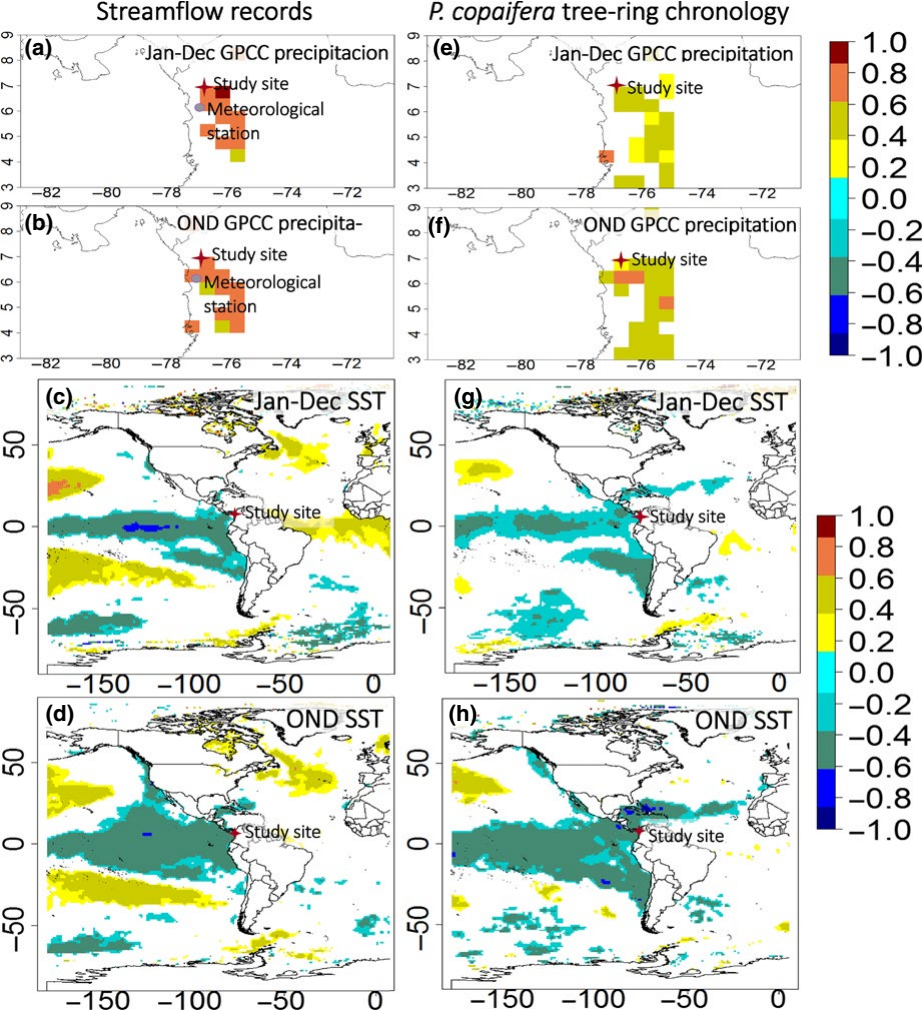
Spatial correlation from 1965 to 2002 between: streamflow and GPCC V6 0.5 gridded precipitation annual spanning from January–December (a) and for October–November–December, OND (b); the *P. copaifera* chronology and GPCC V6 0.5 gridded precipitation annual (e) and for OND (f); the streamflow records and HaddlSST1 annual (c) and for OND (d); the *P. copaifera* chronology and HaddlSST1 SST annual (g) and for OND (h). Note that only significant correlations (*p* < .10) are plotted

For the period from 1965 to 2002, spatial correlations between streamflow records and annual gridded precipitation from GPCC V6 0.5° show that the streamflow in the low Atrato River is significantly and positively related to upstream watershed precipitation (Figure 6a,b) and negatively correlated with SSTs over the western Pacific region (Figure 6c,d). The same significant positive relationships were also found between the *P. copaifera* chronology and the gridded precipitation from GPCC V6 0.5° in the upper part of the river basin (Figure 6e,f), with more intensity (higher correlations) with the seasonal average OND corresponding to the last months of the rainy season (Figure 6f) than annually (Figure 6e). Field correlations between our chronology and SSTs (Kaplan et al., 1998) for the same period show negative correlations with SSTs across the tropical Pacific Ocean and the Caribbean (Figure 6g), also stronger for the OND period (Figure 6h), exhibiting a very similar pattern that field correlations between streamflow and SSTs (Figure 6c,d).

### 3.3 Radiocarbon analysis

**Figure 7:**
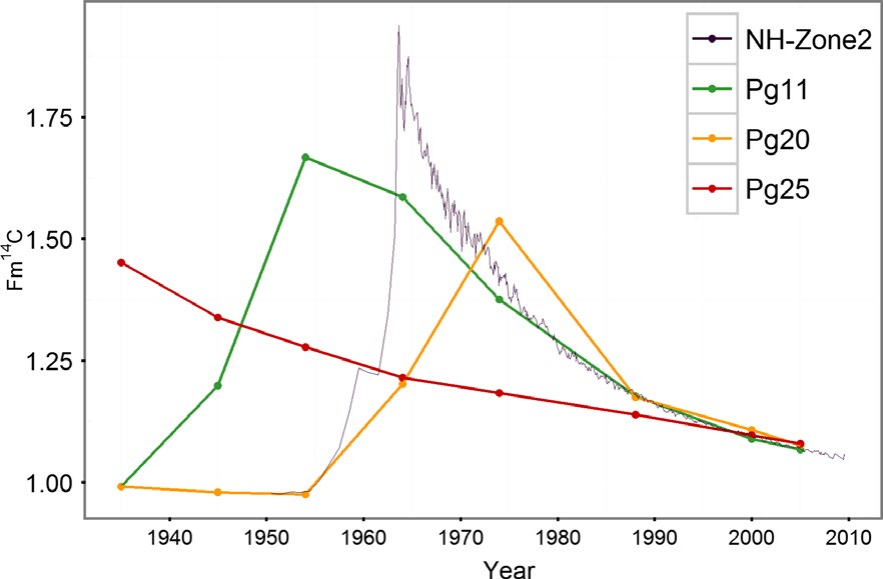
The fraction modern carbon (Fm^14^C) values of the analyzed samples of *Prioria copaifera* selected from three different trees (Pg11, Pg20, and Pg25) are plotted against the atmospheric record compilations for NH‐Zone2 from 1950 to 2010 (Hua et al., 2013)

Sixteen of the twenty four ^14^C measurements done in this study from three different trees show significant differences with the calendar dates obtained using dendrochronological methods, whereas eight of twenty‐four show good agreement with the NH‐zone 2 ^14^C curve (Hua et al., 2013; Figure 7). Best agreements were found for Pg11 and Pg20 trees, mostly during the recent period (e.g., from 1975 to 2006). Nevertheless, all tree rings analyzed with dendrochronological ages before 1975 differed significantly from the atmospheric ^14^C levels expected, indicating that the chronology becomes significantly weaker from this year backward. This also implies that for some years more than one ring was detected and measured using the standard dendrochronological techniques described in the methodological section, and therefore a cumulative effect in calendar ages is observed. For the period from 1962 to 1980, an excess of 4.25 ± 2.9 annual tree rings were identified per decade in Pg11, resulting in a maximum mismatch of 17 rings by 1962. The Pg25 ring‐width series resulted in 5.4 ± 2.2 misidentified tree rings per decade from 1973 to 2005, resulting in an offset of approximately 40 years by 1973. Finally, in Pg20 four tree rings were not identified from 1988 to 1974 and three additional rings were detected from 1974 to 1964, resulting in a cumulative mismatch of seven rings by 1964. Note that these intra‐annual growth bands presenting a subannual resolution were anatomically indistinguishable from annual rings during the macroscopic evaluation.

### 3.4 Instability in the relationships between *P. copaifera* growth and environmental variables

**Figure 8:**
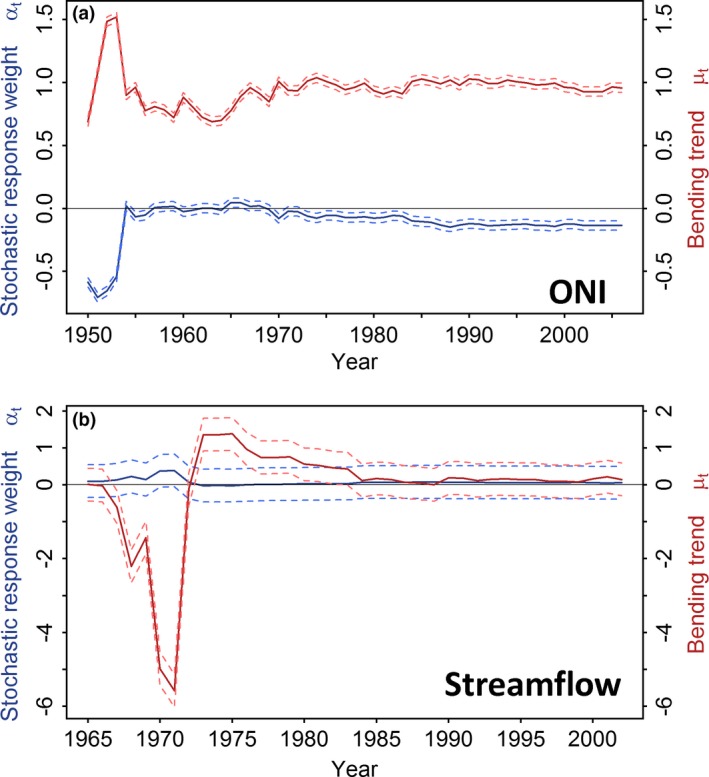
Stochastic response function estimates, derived from a structural time series model, between the tree‐ring chronology and the Oceanic Niño Index (ONI) from 1950 to 2006 (a) and streamflow from 1965 to 2002 (b). Red lines show the estimated integrated random walk trend μ_t,_ and the blue lines shows the response of α_t_. Both relationships were time dependent until 1980, and become stable from 1980 to 2006. Dotted lines denote 95% confidence limits

The correlations between the ring‐width chronology and annual streamflow, temperature, ONI, PDO, and SOI were higher when a shorter period from 1980 onwards (Table 2) was used instead of the maximum time span available for these records (Table 1). In agreement, the STMs calculated for streamflow and ONI (i.e., highest correlations) as dependent variables of growth, which explain 97% of the variance for streamflow and 21% for ONI, confirm these results (Figure 8). Significant relationship between growth and these climatic records is occurring when the zero value falls outside the dotted line for the stochastic response weight α_t_ (blue lines in Figure 8). The growth–climate relationship shows an early period of instability in both estimators α (stochastic response weight) and μ (the bending trend) that become constant from about 1980 onwards (Figure 8). For the growth–ONI relationship, both α_t_ and μ_t_ became stable and significant approximately at 1980 and stayed stable until the last year, 2006 (Figure 8a). For the growth–streamflow relationship, α_t_ was stable from 1970 to 2006 and μ_t_ became stable after 1980, although none of them were significant probably due to the short time span of the streamflow records (Figure 8b). Overall, we detected a stable response after 1980 for both variables ONI and streamflow explaining *P. copaifera* growth.

## 4 DISCUSSION

Although the *P. copaifera* ring‐width chronology generated in this study passed all the standard dendrochronological quality tests (successful crossdating, EPS > 0.85 and significant correlations with climate), the ^14^C results demonstrated a lack of annual tree growth periodicity in three of the trees composing the chronology (Figure 7).

The significant disagreement between the dendrochronological dates and the ^14^C atmospheric signal of the tree rings of *P. copaifera* cannot possibly be attributed to incomplete removal of extraneous substances during chemical pretreatment. Our holocellulose extraction procedure is robust, and has been proven to produce a perfect tree‐ring/^14^C crossdating match in subtropical tree species during the bomb peak period, when 71 consecutive tree rings were processed and measured (Santos et al., 2015). In addition, the targets produced here from the tree Pg25 were processed and ^14^C‐AMS measured together with another set of postbomb tropical wood samples independently validated by us that shows perfect annual frequency of tree‐ring formation (Andreu‐Hayles et al., 2015).

Problematic anatomical features, such as vague, discontinuous, missing, and false rings are very common issues in tropical species, and it is possible that they may hinder the correct identification of tree‐ring boundaries (Brienen & Zuidema, 2006). However, we believe that the large offset in the ^14^C dates observed in our study is mostly due to the presence of intra‐annual parenchyma band formation that leads to rings with sub‐annual resolution. Therefore, *P. copaifera* trees may have produced one or more rings per year, without a constant frequency, as suggested before by Grauel (2004). The formation of sub‐annual rings could be the response to strong intra‐annual variability in growth during a given year. In that case, it would be impossible to anatomically differentiate annual rings from sub‐annual rings in years when the limiting factors (e.g., streamflow in *P. copaifera*) vary substantially within a given year.

The formation of fewer subannual rings, specially over the recent period, could be due to the age of the trees: older trees have lower rates of intra‐annual structure formation (Dünisch, Ribeiro Montóia, & Bauch, 2003); stand/crown position: trees under suppressed growth conditions tend to form more intra‐annual structures (Groenendijk et al., 2014); and/or physiological factors as root depth or less susceptibility to insect attacks (Copenheaver, Pokorski, Currie, & Abrams, 2006). Our results imply some important limitation to use the *P. copaifera* ring‐width chronology for reconstructing the climate along the low Atrato River. The phenology of those trees in the low Atrato River environment makes reliable chronologies very difficult to be developed, but it does not preclude a potential annual pattern when this tree species is growing under distinct environmental conditions. Nevertheless, the annual nature of *P. copaifera* tree rings was previously reported by McKenzie (1972) using the periodic cambial wounding technique, as well as by Herrera and del Valle (2011) and Giraldo and del Valle (2011) by examining ^14^C signature in a single ring of the recent period. This alerts about the danger of generating inaccurate climate reconstructions in the tropics (Herrera & del Valle, 2011) if a thorough independent validation of the annual nature of the tree rings is not performed.

Although obtaining multiple high‐precision ^14^C‐AMS dates is still expensive, it is a good approach to corroborate the annual nature of tree rings for species with challenging anatomical features defining growth layers, never tested before with dendrochronological methods (Andreu‐Hayles et al., 2015; Santos et al., 2015), and/or are growing in regions with a complex seasonality. Measurements before and after the bomb spike can be taken in order to obtain a precise assessment of the annual tree‐ring identification over the entire chronology. Besides the ^14^C analyses, although more time‐consuming, there are other independent validation procedures that can be considered such as periodic dendrometric measurements (Callado, Roig, Tomazello‐Filho, & Barros, 2013), periodic cambial wounding (Mariaux, 1967), successive microsampling for evaluations of cambial activity throughout a given period (Krepkowski, Bräuning, Gebrekirstos, & Strobl, 2011), or counting rings in trees with known ages (Dünisch et al., 2003).

Despite the discrepancies between the dendrochronological dates and the ^14^C dates, the climate signals detected in the tree‐ring chronology seem to be plausible and not accidental, providing insights of the sensitivity of *P. copaifera* growth to climate. Streamflow was found to be the most important environmental factor limiting *P. copaifera* growth (Figure 5a). We initially hypothesized that the seasonal extended floods would limit growth due to anoxic conditions (i.e., negative relationship), but we found that floods (high‐streamflow periods) promoted growth for this species (i.e., positive relationship). Thus, wider rings were found under high streamflow conditions, and vice versa. Streamflow is driven by precipitation over the upper watershed (Figure 6a,b) that is associated with lower SSTs over the Pacific region (Figure 6c,d) as described before by Poveda et al. (2001). *P. copaifera* growth is also correlated with climate indices linked to ocean and atmospheric patterns across the Pacific such as ONI, SOI, and PDO (Figure 5) that is coherent with the negative relationships with tropical Pacific SSTs (Figure 6g,h) and positive relationship with gridded precipitation over the watershed (Figure 6e,f). ENSO is associated with higher than normal precipitation and streamflow over the entire watershed during La Niña events, but drier than normal conditions during El Niño events (Poveda, 2004). The ONI, SOI, PDO, and SST signatures in the *P. copaifera* tree rings confirm that ENSO variability may promote larger tree rings during La Niña years; and narrower rings during the El Niño events. Clark, Piper, Keeling, & Clark (2003) and Grauel (2004) reported that *P. copaifera* diminished during the El Niño of 1997–1998 and increased during the La Niña period of 1999–2000. This pattern was reported in flooded stands similar to our study site, but also in more diverse and drained forests, supporting the climate–growth relationships described here.

Although spurious significant correlations cannot be discarded as the cause for the detection of hydroclimate signals in the *P. copaifera* chronology, other feasible explanations may be suggested. We hypothesize that during the most recent years correlations over the correct calendar year between the chronology and climate series, or just lagged 1–2 years, may have occurred because: (1) the good agreement between the ^14^C results from Pg20 and Pg11 trees and the ^14^C curve NH‐zone 2 (Hua et al., 2013) from 1980 onwards (Figure 7); (2) higher correlations between the chronology and instrumental records from 1980 onward than during the entire available period (Tables 1 and 2); and (3) the stability in the relationships between the chronology and both streamflow and ONI from 1980 to 2006 (Figure 8). These evidences suggest a correct identification of annual tree rings during the last decades. Therefore, considering that the climate series cover the last 24 or 37 years, the climate signal found is likely to be the result of correlations between climatic time series and the *P. copaifera* chronology mostly with correct calendar years.

## 5 CONCLUSIONS

In dendrochronology it is common practice to assume annual periodicity without further testing when: (1) there is common tree growth variability shared among trees; (2) significant correlations with meteorological records with annual resolution are found. Here, we show how these criteria are not valid for trees of the tropical species *P. copaifera* Griseb., located at the Atrato River floodplain in Colombia (7°20′N, 77°57′W). Despite that the *P. copaifera* chronology crossdated well and exhibited strong and coherent climatic signals, these evidences were not reliable proofs of the annual nature of tree‐ring formation due to the presence of intra‐annual growth bands not anatomically distinguishable from annual rings. The wide range of results reported by different dendrochronological studies in relation to the ^14^C analysis lead to the recommendation to conduct multiple ^14^C measurements per tree on wood tissues belonging to the sharp ascending and descending portions of the ^14^C bomb curve when attempting to corroborate the annual nature of the tree‐ring formation, and in more than one tree if possible. It is also advisable to test the stability of the climate signal recorded by the tree‐ring chronologies before the development of climate reconstructions or other applications requiring high‐precision dating. Larger sampling replication, different sampling sites, and longer reliable climatic records may also aid toward the generation of more reliable chronologies and climate reconstructions in tropical regions.

## CONFLICT OF INTEREST

None declared.

## AUTHOR CONTRIBUTION

D.H.R. and J.I.V generated the tree‐ring width data. G.S. generated the radiocarbon data. D.H.R and L.A.H. designed the study (including data analyses) and wrote the manuscript. D.H.R. performed data analyses. P.G. supervised the climate component. All authors contributed to analysis interpretation and manuscript development.

## DATA AVAILABILITY

The data used in this study are archived at the Forest and Climate Change Laboratory of Universidad Nacional de Colombia, please contact daherrerr@unal.edu.co or jidvalle@unal.edu.co.
